# Analysis of workload generated in the two years following first consultation by each new cancer patient: studying the past to plan the future of cancer care

**DOI:** 10.1186/s12913-022-08573-3

**Published:** 2022-09-21

**Authors:** SK. Garattini, F. Valent, AM. Minisini, C. Riosa, C. Favaretti, L. Regattin, G. Fasola

**Affiliations:** 1 Department of Oncology, Academic Hospital of Udine ASUFC, Piazzale Santa Maria della Misericordia 15, Udine, UD 33100 Italy; 2 Institute of Hygiene and Clinical Epidemiology, Academic Hospital of Udine ASUFC, 33100 Udine, UD Italy; 3 Center for Leadership in Medicine, Catholic University of Sacred Heart, 000168 Rome, RO Italy; 4 Medical Director, Academic Hospital of Udine ASUFC, Piazzale Santa Maria della Misericordia 15, Udine, UD 33100 Italy

**Keywords:** Oncology workload, Oncology workforce, Oncology planning, Sustainability

## Abstract

**Introduction:**

Prevalence of cancer patients is dramatically increasing. We aimed at quantifying the oncology workload generated by each new cancer patient in the two years following first consultation.

**Methods:**

In this record-based retrospective study, we retrieved data of all newly diagnosed patients treated at the Oncology Department of Udine Academic Hospital between 01.01.2012 and 31.12.2017. We calculated mean number and standard deviation of the activity type generated by each new cancer patient during the following 2 years.

**Results:**

Seven thousand four hundred fifty-two cancer patients generated a total of 85,338 clinical episodes. The two-years mean number of oncology episodes generated was 11.31 (i.e., for every 1,000 new cancer patients, 11,310 oncology activities are generated overall in the following two-year lapse). Patients with advanced disease generated the highest workload (24.3; SD 18.8) with a statistically significant difference compared to adjuvant and follow-up patients (*p* < 0.001). The workload generated in the period 0–6 and 0–12 months was significantly higher than in the following months (*p* < 0.001) and it was also higher for patients initially designated to treatment (*p* < 0.001).

**Conclusion:**

This is the first study reporting on the mean oncology workload generated during the 2 years following first consultation. Workload is the highest for patient with advanced disease, especially in the first months and in patients in active treatment. A detailed analysis of workloads in oncology is feasible and could be crucial for planning a sustainable framework for cancer care in the next future.

## Background

Cancer represents the second leading cause of death in western countries [1] and the number of cancer patients is increasing over the last decades due to many factors of improvement. Cancer statistics report of year 2021 indicates that there will be 1,898,160 new cancer cases in USA [2]. The same report calculates a 31% reduction of cancer mortality from 1991 through 2018 [3]. These are exciting advances in cancer care but the growing prevalence of patients imposes a reflection for the present and for the next future on the consequent social, managerial and economic burden. Financial resources as well as human resources are lacking (both for physician and nurses). Luego Fernadez R. et al., reported that cancer care cost 126 billion euros in 2009 with the highest costs for lung cancer, followed by breast and colorectal cancer [4]. A survey from American Society of Clinical Oncology (ASCO) describes a warring reduction in number, and an increase in the size, of practices across USA form 2013 through 2017 [5]. ASCO also created a task force with the aim of analyzing the workforce of oncology care professionals across US. In its 2020 document it is reported that medical oncologists practicing on the US territory have worryingly decreased and the number of oncologists under 40 years of age represented 12,7% of the population of American oncologists, while almost 20% were over 64 [6]. In a report from Fundytus et al., a more warring overload of activity is described for physician oncologists working in low-middle income countries [7]. It is not clear yet how much oncology departments worldwide are suffering for an overload of activity. Only few publications tried to assess the workload issue in Medical Oncology and mostly reported only the results of international surveys [8, 7, 9]. Anyway, this issue has never been systematically dealt with.

In order to program the future need for cancer care, it is necessary to quantify and analyze the workload actually faced by cancer departments. Therefore, we conducted a retrospective single institution study aimed at determining the burden of oncology activities generated over the following two years by each new cancer patient taken on charge by our Oncology Department.

## Methods

### Study design and aims

This is a one-institution retrospective study conducted at the Oncology Department of the Joint Commission International accredited Udine Academic Hospital. We collected administrative data on the number and type of oncology clinical episodes (the burden of clinical activities, called workload) generated in the two years following first consultation by each newly diagnosed cancer patient taken on charge. We retrieved data on our patients using the Health Information System of Friuli Venezia Giulia Region. It is a repository for epidemiological use, managed by Insiel S.p.A. (34,133 Trieste, Italy), the software house of the Friuli Venezia Giulia Region, and including several regional health related databases. Such databases can be deterministically linked with one another at the individual patient level through an anonymous stochastic univocal key. For the present study, the database of oncology charts was analyzed. Despite all data were anonymous and patients could not be identified, all records regarding a single patients could be identified through the univocal patient stochastic key. The objective of the study was to calculate the workload generated by each new cancer patient in the two years following first consultation. We included in the study all consecutive patients who have had an initial first consultation in the period between 01.01.2012 and 31.12.2017. Patients without a second clinical episode within 12 months were excluded. Follow-up was collected up to 31.12.2019 (8 years)**.**

### Study setting

The research was fully conducted at Academic Hospital of Udine (Italy) in the Oncology Department which is devoted to the cure and the research on cancer. Our department is located in the main highly specialized Hub Hospital of Friuli Venezia Giulia Region, north eastern Italy, that is administratively responsible for an area of about 600,000 inhabitants but, being the main hospital of the region, it functionally serves a population of about 1,200,000 inhabitants. The Academic Hospital hosts the Udine University School of medicine and our structure is devoted to advanced oncology care and to academic as well as sponsored research. We collect international and national studies ranging from phase II to phase III clinical studies in the field of breast cancer, lung and thoracic cancers, gastro-intestinal cancers, genito-urinary cancers, melanoma, sarcomas and rare cancers. Last, we are also responsible for innovative cancer care in the field of immunotherapy and tumor molecular board development. Our institution operates within the frame of the Italian National Healthcare system (SSN). SSN is a highly decentralized, region-based system, that provides coverage for authorized health services to all Italian citizens and to foreigners who are recognized as residents of the country.

### Statistical analysis

Oncology activities were classified, according to the Italian Oncology Associations AIOM-CIPOMO guide lines [10]. This is a consensus document published in order to standardize Italian oncology activities categorizing them as follows: first consultations (60’-90’), pre-treatment visits (20’), unplanned presentations (30’-90’), hospitalizations, re-assessments (30’-45’), follow-up visits (20’-30’) and inpatient oncology advices. We calculated the mean number and standard deviation for all of the activities generated by each new patient during the two-years following the first consultation. After collecting the whole information, we examined the activity load generated by three distinct categories of patients individuated according to initial setting of care: follow up, adjuvant treatment and advanced disease. We also analyzed data according to the timing from first consultation (0–6, 0–12, 13–24, 7–24 months). All the analyses were conducted using SAS v9.4 (SAS Institute Inc., Cary, NC, USA).

### Ethical statement

All procedures contributing to this work comply with the ethical standards of the relevant national and institutional committees on human experimentation and with the Helsinki Declaration of 1975, as revised in 2008. No Ethics Committee approval or informed consent were required in Italy since the data used in this research were completely anonymous with very low possibility to identify patients.

## Results

### Demographics

We obtained retrospective data on oncology workload derived from 7,452 newly diagnosed consecutive cancer patients (pts) (Table 1). Median age at first consultation was 66 years (IQ range 56–74). The population of the analysis was composed by 58.2% of female (4,336 pts) and 41.8% male subjects (3,116 pts). At first consultation: 25.5% of the patients were taken on charge for follow up (1,903 pts), 41.4% for adjuvant treatment (3,086 pts) and 33.1% of the patient were referred for the treatment of advanced disease (2,463 pts). In our series, 32.6% of patients presented with breast cancer (2,431 pts), 25.8% with gastro-intestinal cancers (1,925 pts), 13.9% with lung cancer (1,037 pts), 3.5% with prostate cancer (260 pts) and 24.1% with other type of cancers (1,799 pts), proportions that can be considered well aligned with the epidemiology of European Countries [11].

**Table 1:** Demographics main characteristic of the 7452 patients that have been analyzed. Values are expressed with mean with its interquartile range (IQ), absolute number and percentage

Total pts 7452	
**Age**	66 (IQ range 56–74)
**Gender**	
Male	3116 (41.8%)
Female	4366 (58.2%)
**Initial intention**	
Follow up	1903 (25.5%)
Adjuvant	3086 (41.4%)
Advanced	2463 (33.1%)
**Type of Cancer**	
Breast	2431 (32.6%)
Gastro-intestinal	1925 (25.8%)
Lung	1037 (13.9%)
Prostate	260 (3.5%)
Other type	1799 (24.1%)

### Workload in the two years following the first consultation

Within the time frame in analysis, the Oncology Department of Udine dealt with a total number of 93,098 clinical episodes (including first consultations). All these activities were analyzed for this study. First consultations accounted for 8.3% of the workload. Excluding first consultations, 52.8% of the activities was dedicated to pre-treatment visits, 17.6% to follow up visits, 13.8% to re-assessment visits, 8.9% to unplanned presentations (managed in a clinic dedicated to oncology urgencies), 3.7% to hospitalization of acute patients, 3.2% to consultancies for inpatients of other wards (Table 2).

**Table 2:** Mean number of activities generated in the following 2 years by each new cancer patient. ^a^First consultations are excluded. Numbers are indicated with mean, standard deviation (SD) and total number of events (E)

**Total pts** 7452	**Total Mean (SD)**	**Total N of visits**
**Mean Number of Oncology Activities (sum)**	**11.31**	**E 85,338**^**a**^ **(100%)**
Pre-treatment visits	5.99 (8.75)	**E 45,095** **(52.8%)**
Follow up visits	1.93 (1.86)	**E 15,001** **(17.6%)**
Re-assessments	1.60 (1.27)	**E 11,753** **(13.8%)**
Urgencies/Unplanned presentations	1.01 (2.15)	**E 7,631** **(8.9%)**
Hospitalizations	0.42 (1.20)	**E 3,146** **(3.7%)**
Inpatient Oncology advice	0.36 (0.83)	**E 2,712** **(3.2%)**

Analyzing all the activities carried out following first-consultations (85,338 single episodes) we derived that a new cancer patient generates a mean of 11.31 activities during the two years following first access (Table 2). The mean number of treatment visits was 5.99 (SD 8.75). We calculated a mean of 1.93 (SD 1.86) follow up visits, 1.60 (SD 1.27) re-assessments, 1.01 (SD 2.15) unplanned presentations, 0.42 (SD 1.20) hospitalizations and 0.36 (0.83) in-patient consultancies (Table 2).

In our analysis, a patient who has a follow up program at first consultation, generates a mean number of activities corresponding to 5.18 (SD 4.0) during the following 2 years (Table 3); a patient with adjuvant treatment program generates 14.8 (SD 13.5) visits while a patient classified as having advanced disease generates a mean of 24.3 (SD 18.8) subsequent visits. The mean workload generated by each case is statistically different in the three groups (*p* < 0.001). Starting treatment for advanced disease produces in our experience the highest workload, followed by adjuvant therapy and follow up program respectively.

**Table 3:**
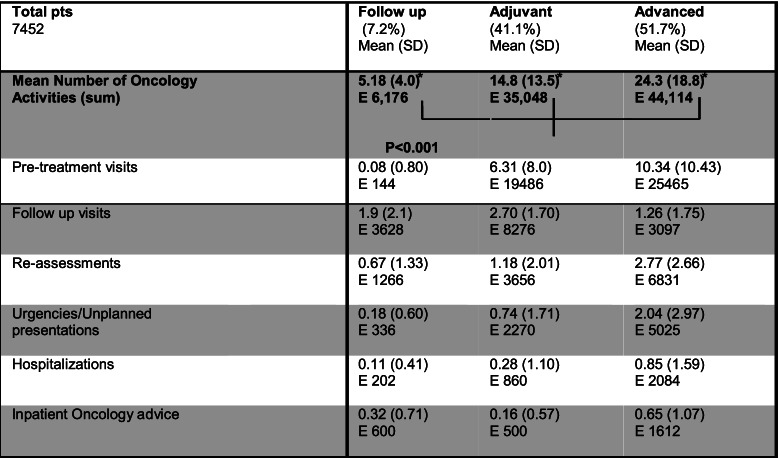
Mean number of activities by initial intention of treatment. Numbers are indicated with mean, standard deviation (SD) and total number of events (E)

Relying on these results it could be feasible to estimate the amount of activities that would be generated every 1,000 patients in an average Oncology Department**.** Indeed, 11,310 total clinical activities would be generated by 1,000 new cancer patients, in the following 2 years, independently from the initial program assigned at the first consultation. This amount is subdivided into 5,180 episodes generated by patients in the follow up setting, 14,800 by adjuvant patients and 24,300 by advanced disease patients.

### Two-year workload according to time from initial visit and to treatment program

The mean number of activities generated during the first six months was 8.08 (SD 7.49) compared to 4.66 (SD 5.46) activities generated from 7 to 24 months after the initial visit (Table 4). During the first 12 months the mean workload for each case was 11.46 (SD 11.10) while it was 6.33 (8.57) in the month from 13 through 24. Of note, the reveled differences were all statistically significant (*p* < 0.001 for 0–6 months vs 7–24 months; *p* < 0.001 for 0–12 months vs 13–24 months).

**Table 4:**
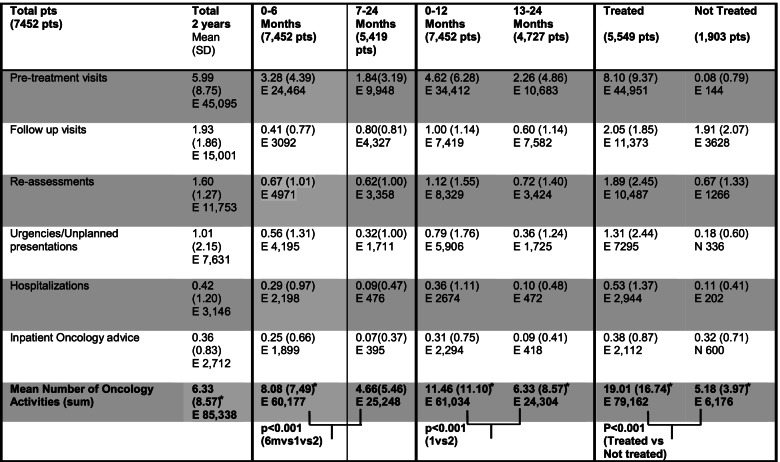
Median number of activities sorted by time from initial consultation (0–6 months after vs 7–12 months after, or 0–12 vs 13–24). Comparison is also made between patients who have received treatment vs the ones who had follow up only (treated vs not treated). Numbers are indicated with mean, standard deviation (SD) and total number of events (E)

Mean workload for the patients with a treatment program was 19.01 (SD 16.74) episodes each compared to 5.18 (SD 3.97) of the other ones, again with a statistically significant difference between the two groups (*p* < 0.001)**.**

## Discussion

Over the last decades Oncology has witnessed an unprecedented capacity of prolonging life of cancer patients due to many factors of improvement such as the advent of immunotherapy and target treatments for advanced diseases [12–19]. All this translates into a growing cancer prevalence that imposes serious reflections on the future sustainability and organization framework of cancer care delivery. An estimate of cancer incidence in US depicts a scenario of growing trends of prevalence for breast cancer, followed by melanoma, lung cancer and colorectal cancer [20]. Many international studies, including some published by ASCO (American Society of Clinical Oncology), have tried to quantify the resource utilization and costs in some specific oncology fields such as radiotherapy, breast cancer, lung cancer, colorectal cancer, prostate cancer, gastric cancer, bladder cancer and other [21–28]. Moreover, a warning comes from the future shortage of nurses and physicians dedicated to the discipline [16]. Then the question to be raised is: will decision makers, both on political and professional side, be able to redesign an appropriate efficient system of cancer care delivery in the very next future? First step for programming the future would be to quantify oncology workforce needs but such reports are substantially lacking. Some previous reports have tried to estimate the burden of workload on oncologists, mainly by the means of “snowball” international surveys [7, 9]. It is to be pointed out that most of these projections come from America.

In 2012 our research group published a study by Fasola G. et al. that proposed a model to calculate the need of human resources in Oncology [29]. In that report, considering only patients on active treatment, it was calculated that during 2006 each patient generated an average of 16 clinical evaluations, which translated into 8 and 16 h of physician and nurse working time, respectively. The authors also estimated that an oncology department would be in the need of one physician and three nurses for every 200 novel patients taken on charge.

Moving from these preliminary data, in this record-based single-institution retrospective study, we aimed at estimating the oncology workload generated by each newly diagnosed cancer patient in the two years following first consultation to obtain an overview of the present situation and being able to estimate future needs in terms of resources. A 2-year (after first consultation) observation period was set because it is the frame of time in which cancer care faces the highest workload due to the diagnostic effort of the initial phase, the highest probability of relapse for follow up/adjuvant treatment patients and because the most of the aggressive advanced cancers have a median OS of less than one-two years. We have analyzed a total number of 7,452 cancer patients that have generated 85,338 clinical episodes (over an 8-year period). Such refined retrospective data were retrieved thanks to a dedicated IT system set up in 2001 as computerized physician order entry (CPOE) and, since 2004, as complete electronical medical record (EMR). The long observation time, and the number of cases observed, allow us to collect more reliable information. Last, our workload calculation has been possible because during our observation time no major changes have occurred in national and regional healthcare rules, hospital or departmental care delivery organization. Moreover, our model is based on a calculation made on a stable care path (from diagnosis to treatment) and it is unlikely that few cases taken on charge from other hospitals in the middle of their cancer journey could impact the present conclusions.

We found that most patients were referred for adjuvant treatments (41%) followed by the ones taken on charge for advanced disease (33.1%) and for follow up (25.5%). The activity load partition by cancer type resulted as follows: about 30% breast cancer, 26% gastro-intestinal cancer, 14% lung cancer, 3.5% prostate cancer and 24% other cancer types. Taken together almost 60% of the visits are dedicated to breast and gastro-intestinal cancers. Taking into account the tumor type distribution, our case series can be considered well representative of the Italian cancer patients population as reported by the AIRTUM (Associazione Italiana Registri Tumori) Italian Registry of cancers [30]. We believe that this corresponds to what could be found in an average European Oncology Department elsewhere. Analyzing the type of activities our oncologists were dedicated to, we found that half of these (about 50%) were constituted by pre-treatment medical visits but a not negligible 16% of episodes were follow up visits (15,001 overall). This aspect is highly relevant considering that, for instance, sharing this type of non-highly specialized visits with general practitioners could create time and spaces for more specialized activities such as treatment visits or re-assessments. Noteworthy, 8% of unplanned presentation reflects a very specific activity, not uniformly developed in Oncology Departments, that consists of a clinic for “oncology urgencies”. In the subsequent analysis performed, we compared the workload generated by each new cancer patient according to initial setting of disease: follow up, adjuvant or advanced disease. We found that a patient in advanced setting of disease generates a sevenfold higher workload compared to a follow up patient while an on-adjuvant treatment patient an almost sixfold higher burden. This information could allow a more appropriate allocation of resources, on the bases of the setting of care, in the frame of a “Comprehensive Cancer Care Network” made of hospitals with relatively higher or lower volumes of activity. Last, we performed an analysis of workloads in the first 6, 12 and 24 months. We found that the first 6–12 months after first consultation result in significantly higher workload. This remark would allow policymakers to invest resources accordingly.

This system of reporting has been successfully used by our department, over the last five years, to negotiate the hiring of new human resources at hospital and regional level. Our requests have been legitimated by this reliable and independent report of the need of resources (usually hiring are made worldwide upon subjective claims made upon impressions of overload). It would be thus exportable to other institutions that may calculate their needs upon a 1,000 new patients loads estimate. Moreover, our study poses the basis for workload calculation that could be useful in many different contexts. Indeed, it is well known that low-middle income countries, compared to high income countries, suffer from oncology activity overload [7, 31]: our calculation method could serve as a useful tool for those developing countries that will face a fast changing cancer epidemiology over the next years. The issue of medical workload is also perceived crucial in other specialties dealing with diseases with incremental epidemiology [32–35]. It is also reasonable to propose that, given this method, our system of analyses could apply to other departments dealing with chronic conditions such as cardiology, pneumology, nephrology and potentially any other specialty.

This study presents also some limitations: our report focuses on a single institution experience even though representative of an average Oncology Department of a western hub Hospital. We are aware that such a large retrospective study could not probably be replicable in a multi-institutional retrospective manner considering the lack of adequately comparable IT systems. Moreover, it has to be considered that decision makers could show different approaches in measuring volumes of activity in oncology through a proper IT system. Among other limitations we discuss that at this level, given the managerial source of data, we could not granularly retrieve the patient-level factors that may contribute to generate oncology workload; moreover there are other factors requiring personnel and resources in the most complex diseases (i.e. surgery, radiotherapy, biliary drainage positioning and other..) that could be incorporated in the calculation in the case of broader availability of data and wider research interests. Unfortunately, all these further calculations were beyond the aim of the study even if they add complexity and generate a broader concept of oncology workload that could be investigated but extend beyond the aims of the sole Oncology Department. Despite these caveats, our research group is committed to deeper understanding of the phenomenon of growing oncology workload: indeed we are planning to retrieve more details from a patient level analysis and to analyze differential loads by cancer type. As to external reproducibility, it would be very interesting to compare our results about workloads with other studies from international institutions. Nevertheless some strengths can be identified in our study. First, it offers an estimation of Oncology workload as a quantitative report, which is not so common in Europe. Second, our data come from the Italian, Beveridge-like, Healthcare System, factor that, in the opinion of the authors, excludes the biases related to generating workloads on the bases of convenience and costs. Moreover we think that in such a Beveridge-like system potentiating primary and secondary prevention could be useful strategies aimed at reducing the expected oncology workload. Third, we were able to calculate the load of activity in a registry-based manner, rather than from surveys, thanks to our robust electronic data entry which was adopted as long as in 2004. The volume of medical assistance analyzed in this study (7,452 patients over a six year period with 85,338 total oncology episodes) is very large and, according to the demographics, our disease mix is fully in line with the epidemiology reported in the latest version of AIRTUM [30], which could be considered representative of cancer epidemiology in a Western Country.

Finally, this study is, to the best of our knowledge, the first to address the issue of quantifying the workload of medical oncologists in an average Hub Oncology Department. We believe that our data could be potentially generalizable to a comparable setting; we therefore hope that our effort would be useful for the achievement of two main goals: first, to increase the awareness of Oncology community on the possibility of quantifying workload derived from cancer care and, second, that quantification will be crucial to adequately plan the needs for cancer care in the next future.

## Conclusions

Every new patient taken on charge by an Oncology Department generates a considerable amount of clinical activities in the two years following first consultation. The highest workload is generated by patients accessing to first consultation in an advanced disease setting and patients with an initial plan of active treatment, especially during the first six months. Estimating workload is feasible and necessary for planning future sustainability of Cancer Departments.

## Data Availability

The datasets generated during the current study are not publicly available due to intellectual property of ASUFC and Friuli Venezia Giulia Region but are available from the corresponding author on reasonable request.
